# DITES: A Lightweight and Flexible Dual-Core Isolated Trusted Execution SoC Based on RISC-V

**DOI:** 10.3390/s22165981

**Published:** 2022-08-10

**Authors:** Yuehai Chen, Huarun Chen, Shaozhen Chen, Chao Han, Wujian Ye, Yijun Liu, Huihui Zhou

**Affiliations:** 1 School of Integrated Circuits, Guangdong University of Technology, Guangzhou 510006, China; 2 Research Institute of IC Innovation (RIICI), Guangdong University of Technology, Guangzhou 510006, China; 3 The Research Center for Artificial Intelligence, Peng Cheng Laboratory, Shenzhen 518000, China

**Keywords:** Trusted Execution Environment, SoC, dual core, cryptography, RISC-V

## Abstract

A Trusted Execution Environment (TEE) is an efficient way to secure information. To obtain higher efficiency, the building of a dual-core system-on-chip (SoC) with TEE security capabilities is the hottest topic. However, TEE SoCs currently commonly use complex processor cores such as Rocket, resulting in high resource usage. More importantly, the cryptographic unit lacks flexibility and ignores secure communication in dual cores. To address the above problems, we propose DITES, a dual-core TEE SoC based on a Reduced Instruction Set Computer-V (RISC-V). At first, we designed a fully isolated multi-level bus architecture based on a lightweight RISC-V processor with an integrated crypto core supporting Secure Hashing Algorithm-1 (SHA1), Advanced Encryption Standard (AES), and Rivest–Shamir–Adleman (RSA), among which RSA can be configured to five key lengths. Then, we designed a secure boot based on Chain-of-Trust (CoT). Furthermore, we propose a hierarchical access policy to improve the security of inter-core communication. Finally, DITES is deployed on a Kintex 7 Field-Programmable-Gate-Array (FPGA) with a power consumption of 0.297 W, synthesized using TSMC 90 nm. From the results, the acceleration ratios of SHA1 and RSA1024 decryption/encryption can reach 75 and 1331/1493, respectively. Compared to exiting TEE SoCs, DITES has lower resource consumption, higher flexibility, and better security.

## 1. Introduction

Since 5G and IoT technologies are rapidly developing, information security issues are becoming more and more important for Internet of Things (IoT) edge devices. To secure information transmission as well as processing, a Trusted Execution Environment (TEE) is proposed [1]. In a TEE, the system running on the processor core is divided into two execution environments, the Trusted Execution Environment (TEE) and the Rich Execution Environment (REE), which are isolated. Therefore, the TEE system provides a secure and a non-secure environment for the actual application operating system. We can deploy different applications on the TEE system depending on the security level to ensure that the program runs securely. Currently, many major CPU providers have introduced a TEE, such as ARM’s TrustZone [2], Intel’s SGX [3], and AMD’s SEV [4]. Trustzone uses hardware virtualization and security bit extension technologies to divide hardware resources into secure and non-secure areas. It is a hardware security isolation technology for IoTs SoC. SGX integrates legitimate and secure software to run in an enclave, which can be isolated from illegal and malicious software to enhance the security of software operations. SEV encrypts Virtual Machine (VM) memory to protect it from physical attacks and prevent other VMs and hosts from reading VM memory data. However, their TEE systems are more oriented toward software-level optimization and have not achieved true isolation at the hardware level. Meanwhile, these technologies are rarely optimized for low-power IoT applications and cannot be adapted to such applications by modifying the underlying hardware implementation, so it is urgent to propose an open-source TEE system for the IoT.

The Reduced Instruction Set Computer-V (RISC-V) is an open source reduced instruction set architecture (ISA), which has been widely used to build SoCs for practical applications, such as a RISC-V-based microcontroller unit (MCU) for flexible power-constrained energy-efficient IoT devices [5], an artificial intelligence (AI) processor that extends the convolutional neural network (CNN) instruction set to RISCV ISA [6] and a spiking neural network (SNN) computing platform based on RISC-V for the flexible configuration of network and neuron models [7], etc. With the rapid development of RISC-V, building security systems for TEE on RISC-V processors is one of the hottest topics. Meanwhile, chip design complexity is increasing greatly with the rapid development of integrated circuit (IC) technology and the continuing reduction of manufacturing process nodes. System-on-chip (SoC) is a widely used technology in current chip design, which integrates a variety of Intellectual Property (IP) to achieve a fully functional SoC with a short design cycle time, complex functionality, and high performance. Furthermore, testing and deploying security algorithms on FPGAs can be more efficient [8], meaning that designing the security algorithm as IP in the SoC can effectively improve the efficiency of the system. The combination of a TEE and SoC design will effectively promote the development of TEE security chips for processing secure information in the IoT environment. Furthermore, it is less efficient to implement a TEE using software. Additionally, at the SoC level, since a TEE system has two different execution environments, it is very suitable to use two CPUs to run both environments, and optimizing the isolated architecture of the dual cores will effectively improve the security of the system. Due to the fact that RISC-V is open source, building a RISC-V based dual-core SoC system with TEE security capabilities will effectively improve the efficiency of the TEE system, as well as drive the development of TEE. In order to solve the current TEE SoCs with high resource consumption and improve the cryptographic unit’s flexibility and the security of dual-core communication, in this paper, we design and implement a lightweight and flexible RISC-V-based dual-core TEE SoC system. The contributions of this paper are as follows.

(a) A dual-core, fully isolated multi-level bus architecture is built. The integrated crypto core supports SHA1, AES, and RSA algorithm acceleration, in which the key length of RSA can be flexibly configured into five modes.
(b) Based on Chain-of-Trust (CoT), we design a secure boot process that combines SHA1 extraction program digest and RSA signature verification. Meanwhile, Input/Output Physical Memory Protection (IOPMP) has been designed as a firewall to restrict access to the CPU and the crypto core.
(c) A hierarchical access strategy is proposed for data exchange to ensure secure inter-core communication.
(d) DITES is implemented using a Kintex 7 FPGA and uses TSMC 90 nm to verify SoC viability.

The paper is organized as follows: Section 2 introduces the current research status of TEE-related SoC systems; Section 3 describes the design and implementation of the SoC system proposed in this paper; Section 4 evaluates and analyzes the performance of the proposed SoC; finally, a conclusion is given to explain the strengths and weaknesses of this paper and future research directions.

## 2. Related Work

### 2.1. Relevant Implementations of TEE Systems

Many researchers have proposed related solutions with the rapid development of TEE SoCs. HOANG et al. [9] proposed a TEE hardware SoC using heterogeneous isolation. Meanwhile, they propose a new boot method based on CoT. However, the whole SoC system is in complete security isolation of 32 bit MCU only in the start bootloader (sBL) and Zero Stage Boot Loader (ZSBL) stages of Boot; in addition, there is no secure inter-processor communication (IPC) channel established between the two cores. They also [10] proposed a TEE-compatible RISC-V system with Cryptographic Accelerators (CA) and used Physical Memory Protection (PMP) to restrict processor rights. The throughput of the signature process using SHA-3 acceleration for large data blocks is higher by 2.5 percentage points compared to the software approach. Kumar et al. [11] proposed ITUS, a secure SoC based on RISC-V. ITUS uses a Memory Protection Unit (MPU) to restrict access to the processor. Meanwhile, the Code Authorization Unit (CAU) and the Key Management Unit (KMU) are used to ensure system security. Jawad et al. [12] proposed a lightweight hardware-based secure boot architecture. The system architecture integrates KMU and CAU. However, the architecture implements fewer security features and does not integrate TEE into the SoC. Dayeol et al. [13] proposed Keystone, the first open-source framework for building custom TEE. Keystone uses simple abstractions provided by the hardware, such as memory isolation and programmable layers under untrusted components (e.g., operating systems). Raad et al. [14] proposed a new secure TEE SoC Secure Architecture (SA), CURE, which is able to provide different types of enclaves and also supports the independent allocation of system resources. Meanwhile, CURE uses firmware technology based on the Security Monitor (SM). However, CURE does not support the acceleration of cryptographic algorithms, making encryption and decryption less efficient. Pascal et al. [15] proposed a heterogeneous CPU architecture for secure RISC-V execution environments in which a separate secure CPU processor is available to provide strong isolation of secure and non-secure domains. The use of a RISC-V secure co-processor provides hardware control over the integrity of the data flow and constrains the access rights to the I/O. Victor et al. [16] proposed Sanctum, a minimally hardware-extended module for strong software isolation based on a RISC-V processor core implementation, which prevents attacks from different software related to domain memory access patterns. Ke et al. [17] proposed the SGX-FPGA, the first FPGA TEE, which secured the communication between the CPU and the FPGA via the PCIe bus protocol to establish a secure hardware isolation path. However, the SGX-FPGA was built as a CPU-FPGA TEE system, resulting in low flexibility. Alessandro et al. [18] proposed a Memory Encryption Unit (MEU) suitable for RISC-V privileged architecture organizations. Their memory encryption design uses the ChaCha cryptographic algorithm, which can enhance the security of RIVC-V cores. However, the architecture is currently not complete with the integration of the PMP. Callum et al. [19] proposed a TEE based on the Physically Unclonable Function (PUF). The PUF-based security module is implemented in an FPGA and interacts with a software platform running in ARM TrustZone on an ARM Cortex core to generate a unique random response for each device, enhancing the security of the system. Armanuzzaman et al. proposed BYOTEE [20], a CPU-FPGA-based framework that can build multiple equally secure TEEs using configurable hardware and software trusted computing bases (TCBs). Meng et al. [21] proposed a scalable RTL-level SoC verification scheme called SEVNOC. It can be used to systematically detect security vulnerabilities in the communication between IPs in SoC designs with NoC architecture. Sushil et al. [22] propose an OTS scheme-based edge infrastructure energy-efficient IoT security architecture to resolve the challenges of smart applications in edge infrastructure.

We have summarized the current TEE-related hardware implementations in Table 1. Complexity indicates the complexity of the CPU used by the TEE SoC, which will directly affect the resource usage of the overall architecture. Flexibility indicates how flexible the cryptography unit used in the TEE SoC is, such as whether the key length is configurable. Secure inter-processor communication (IPC) indicates whether a channel for dual-core secure data exchange is built into the dual-core TEE SoC. Firstly, TEE SoCs offer a higher degree of flexibility compared to CPU-FPGA systems. Second, most currently existing TEE SoCs use complex processors such as Rocket, which requires more resources and is not conducive to promoting TEE SoCs on resource-constrained IoT devices. Third, most of the cryptographic units in current TEE SoCs only support encryption and decryption with a single key length, making the flexibility low. Fourth, the above dual-core TEE SoCs ignore the secure IPC between the dual cores. Finally, only a few works use ASIC implementation to verify the feasibility of the designed TEE SoC architecture.

### 2.2. Requirements of Proposed Work

As can be seen from Table 1, the existing TEE system still has more limitations. Therefore, our proposed system specific implementation requirements are as follows.

(a) Lightweight: The existing TEE SoCs have high processor complexity and require many system resources. Therefore, there is a need to design a TEE SoC system based on a lightweight core processor.
(b) Flexibility: Existing systems are inflexible and cannot handle different lengths of keys. Therefore, there is a need to design secure computing modules compatible with different key lengths to improve the flexibility of system encryption and decryption.
(c) Security: The existing system is not highly secure and does not achieve a completely isolated computing environment for encryption and decryption. Therefore, it is necessary to explore new isolated computing methods and construct a hierarchical access strategy to improve the security of its system for data processing and transmission.
(d) Reliability and validity: Reliability and validity are also important indicators of system performance. Since TEE SoC is a hardware microsystem, FPGA and ASIC technologies need to be used to fully verify the reliability and validity of TEE SoC.

## 3. The Design and Implementation of DITES

### 3.1. Processor and SoC Platform

In our work, we chose to use the T-Head XuanTie E902 [23] developed by T-Head as the CPU. The E902 is compatible with the RISC-V instruction architecture and adopts a hybrid 16/32-bit coding system. The E902 is an extremely low-power, low-cost embedded CPU core, which provides the operating efficiency and performance of a 32-bit embedded CPU at the cost of an 8-bit CPU. It is worth noting that E902 has been widely used in AI accelerators [7,24], industry control MCU [25], and wireless MCU [26]. T-Head has also designed a general-purpose chip design SoC platform based on E902, Wujian100 [23], and it is already being used in industry [27]. Since Wujian100 can only build simple single-core SoC systems, this paper designs a new SoC architecture for building dual-core TEE SoC, which will be described in the next section.

### 3.2. SoC Architecture of DITES

**Figure 1:**
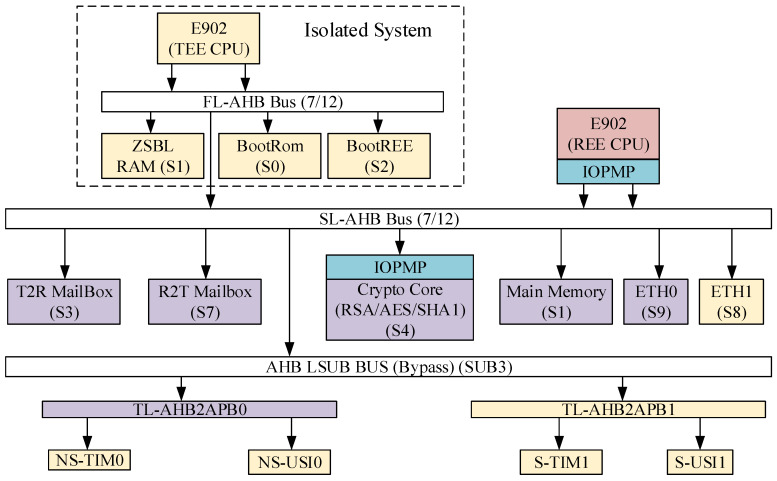
DITES prototype implementation using E902.

The DITES proposed in this paper is shown in Figure 1. The proposed architecture uses two E902 CPU cores; the CPU running in the TEE environment is called the TEE CPU, and the CPU running in REE environment is called the REE CPU. In the SoC architecture, it is divided into the following parts.

(1) **Processors:** The TEE CPU is in an isolated internal system, responsible for SoC security boot-related work and the handling of communication transactions with the REE CPU, such as obtaining ID and encryption and decryption transactions; REE CPU is in an open environment, using IOPMP to constrain the access rights of the REE CPU to achieve secure operation.
(2) **Storage:** There are three main storage units, BootRom, ZSBL RAM, and main memory; BootRom stores the most primitive boot program; ZSBL RAM is responsible for storing the program of ZSBL, and Main Memory is responsible for storing the program running in the TEE and REE environments.
(3) **Communication:** This design uses Mailbox to achieve inter-core communication, and the TEE CPU transmits data to the REE CPU using T2R Mailbox and vice versa using R2T Mailbox.
(4) **Peripherals:** The peripherals mainly include secure peripherals and non-secure peripherals. Secure peripherals include a secure serial port (S-USI1), a secure timer (S-TIM1), and a secure network port (ETH1). Non-secure peripherals include a non-secure serial port (N-USI0), a non-secure timer (NS-TIM0), and a non-secure network port (ETH0).
(5) **Crypto Core:** This architecture contains the hardware implementation of RSA/AES/SHA1, and IOPMP restricts access to this IP.

### 3.3. Secure Hierarchical Bus Architecture

As shown in Figure 1, the dual-core SoC proposed in this paper uses a hierarchical bus design with three layers. The first layer of the bus located in the isolated system is under a secure world called FL-AHB. As the master device, the TEE CPU can access all the devices under this bus, such as BootRom. This layer of the bus does not provide access to devices other than the master device. Therefore, the isolated system is a completely isolated environment, and internal data cannot be accessed directly. The second bus layer is the bus for building the TEE system (SL-AHB), and the TEE CPU can access all of the slave devices on this bus layer. Meanwhile, the REE CPU restricts its access to the slave devices of this bus through IOPMP. The third-level bus is a sub-bus of SL-AHB and is converted to the APB bus to connect the peripheral IP (TL-AHB2APBx), which is restricted to the secure and non-secure APB bus by accessing the ID of the master device. In this design, when the secure boot is completed, both the TEE CPU and the REE CPU will obtain the corresponding code and data from main memory. It is known from the architecture that both master devices have access to the main memory slave devices at the same time, so the AHB Bus with round-robin arbitration used in the architecture makes both CPU cores have the same priority, i.e., the fair occupancy of bus resources.

### 3.4. Design and Implementation of Crypto Core

#### 3.4.1. The Hardware Implementation of SHA1

**Figure 2:**
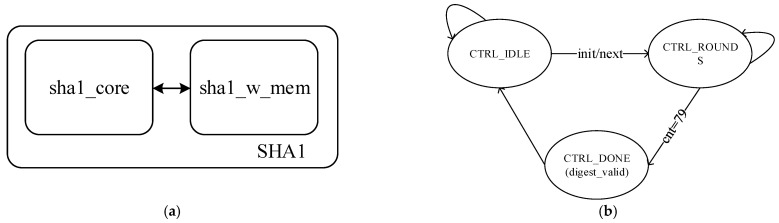
SHA1 hardware unit. (**a**) Hardware architecture of SHA1; (**b**) SHA1 core FSM transfer diagram.

The SHA1 algorithm [28] uses a hash function to compress the message or data into a digest, which enables a message of variable length to generate a 160-bit message digest for verifying that the message has not been attacked or tampered with during transmission. The overall hardware framework of SHA1 is shown in Figure 2a, and the SHA1 hardware acceleration IP hashes the message block obtained after software padding and chunking. **sha1_w_mem** is responsible for the expansion of the message to 32 × 80 bit by a special algorithm:

(1) $$w_{t}=\{\begin{array}{c} M_{t},0\leq t<15\\ ROTL^{1}\left(W_{t-3}⊕W_{t-8}⊕W_{t-14}⊕W_{t-16}\right),16\leq t<79 \end{array}$$

$M_{t}$ represents the sixteen 32-bit words obtained from the input 512 bit data block grouping; *ROTL*^1^ represents the cyclic left shift of 1 bit, and $W_{t}$ represents the data obtained after expansion. The Finite State Machine (FSM) of **sha1_core** is shown in Figure 2b, initialized to the CTRL_IDLE state at reset, jumping to the computation state CTRL_ROUNDS for hashing when the flag signal to start computation arrives, and jumping to the CTRL_DONE state to start the end flag when the 80 rounds of computation are completed. SHA1 can receive 512 bit of data as input. The data is first extended by the sha1_w_mem module. After the extension, the message is sent to the sha1_core for hash operation. After 80 rounds of hashing, we get a 160 bit message digest.

#### 3.4.2. Hardware Implementation of AES

**Figure 3:**
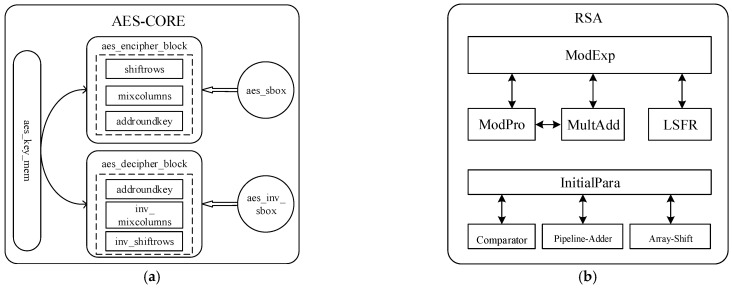
The hardware framework for AES and RSA. (**a**) The hardware architecture of AES module; (**b**) hardware architecture of RSA.

AES is a symmetric encryption algorithm [29], and this paper implements the hardware acceleration of 128 bit, 256 bit Electronic-Codebook-Book (ECB) and Cipher-Block-Chaining (CBC) encryption modes in AES. The overall hardware architecture is shown in Figure 3a. The encryption and decryption process is specified as the key extension according to the length of the key while deciding how many rounds of encryption and decryption are required. For example, for 128 bit, 10 rounds of encryption are required. The first to the ninth round of encryption has the same round function, including four operations: byte substitution, row displacement, column mixing, and round key addition. In Figure 3a, **aes_key_mem** is responsible for key expansion; **aes_encipher_block** is responsible for encrypting plaintext and supporting ECB and CBC encryption modes; **aes_decipher_block** is responsible for decrypting ciphertext, supporting ECB and CBC decryption modes; **aes_sbox/aes_inv_sbox** is responsible for storing sbox/inverse sbox for byte substitution during encryption/decryption, respectively; **shiftrows** plays the role of row shifting during encryption, and inv_shiftrows is its inverse operation; **mixcolumns** plays the role of column-mixing transformation during encryption, and inv_mixcolumns is its inverse operation; **addroundkey is** responsible for round key addition operation. AES can receive 128 bit or 256 bit keys and 128 bit plaintext data as input. The aes_key_mem module first extends the key. After the expansion, the plaintext data is sent to the aes_encipher_block for encryption. The shiftrows, mixcolumns and addroundkey modules are successively used to complete one round of encryption. The number of rounds to be executed depends on the length of the key. AES decryption is the reverse process of encryption, and both of them end up with 128 bits of data.

#### 3.4.3. Hardware Implementation of RSA

RSA algorithm [30] is a very classical asymmetric encryption and decryption algorithm. The core calculation of the RSA algorithm is a large number of modular exponentiations (ModExp). The RSA algorithm needs a modular exponentiation (ME) operation to complete the encryption and decryption operation. To implement the RSA, ME operation must be implemented, that is, ME operation is the basis of RSA. In our system, the RSA hardware unit is designed to support the processing of five different key lengths to improve the flexibility of the TEE SoC encryption and decryption units. Therefore, ME computation also needs to be implemented to support different key lengths. In this paper, we adopt the Montgomery algorithm to simplify the ModExp calculation and use a L-R order-based and base-2 modular multiplication (MonPro) hardware acceleration unit to implement the ModExp. Since each bit of key needs to be verified before each modular multiplication, key cracking can be accomplished by the difference in time and power consumption during encryption and decryption. This means that the current bit of the key is 0 or 1 according to the chip clock and the instantaneous power consumption. For improving the security of RSA hardware encryption and decryption, we add the function of side-channel attack resistance to the ModExp by adding a pseudo-random modular multiplication operation to the base algorithm, and the additional modular multiplication calculations will be added randomly to avoid the cracking of the key. RSA computation with side-channel attacks resistance is shown in Algorithm 1. This algorithm is implemented using a linear feedback shift register (LSFR)-based pseudo-random number generator. The 12 fixed-point decimal form is used, and the pseudo operation of modular multiplication is performed when the generated pseudo-random number is greater than 0.5. The RSA encryption and decryption implemented in this paper can realize the calculation under five key lengths of 192 bit, 256 bit, 512 bit, 1024 bit, and 2048 bit. The hardware IP block diagram of the RSA encryption and decryption IP is shown in Figure 3b. Within the RSA unit, the MultAdd and InitialPara modules are responsible for multiplication, addition, and initialization parameter calculation, respectively. The data of the input of the RSA unit designed in this paper needs to be split in blocks (32 bit), i.e., when the input is 192 bit, it needs to be divided into six 32 bit blocks. Meanwhile, the little-endian is used for data block storage in this paper, i.e., the last 32 bit block is stored in the cache with zero index. Furthermore, the results of the calculation are also stored in the form of blocks. When the calculation is completed, an interrupt is generated to trigger the CPU to read the calculation results.
**Algorithm 1:** Modular exponentiation based on pseudo-random operations1: Given: m, e, and n represent the message, power, and modulus, respectively. 2: Given: r, t, and nprime0 represent the Montgomery parameters, respectively. 3: Given: MonPro(m,t) represents m ∗ t % n. 4: Input: The plaintext m, the key (e, n) of RSA. 5: Output: The ciphertext c after modular exponentiation computation. 6: step1:  $\bar{m}=MonPro\left(m,t\right)$ 7:    $\bar{c}=r$ 8: step2: for i=k−1 to 0 9:      $\bar{c}=MonPro\left(\bar{c},\bar{c}\right)$ 10:      random = *LSFR*(seed) 11:      if $e_{i}=1$ then $\bar{c}=MonPro\left(\bar{c},\bar{m}\right)$ **12:    else if random > 0.5 then**
$\;MonPro\left(\bar{c},\;\bar{m}\right)$      **(no return**
$\bar{c}$**)** 13:     $c=MonPro\left(\bar{c},\;1\right)$
14: step3:  return c

### 3.5. Design of Firewall and Inter-Core Communication

#### 3.5.1. Design of IOPMP

IOPMP provides a protection mechanism for access to devices on the bus. The IOPMP designed in this paper refers to the PMP mechanism in RISC-V architecture, and its specific implementation is as follows.

**Figure 4:**
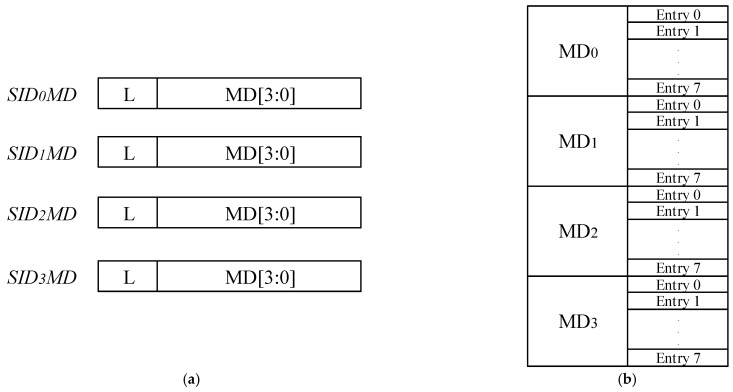
IOPMP internal list distribution. (**a**) IOPMP SIDxMD list; (**b**) list of MD table entries.

(1) As shown in Figure 4, IOPMP has four Memory Domains (MDs), with eight table entries under each MD in our design. Each table entry is designed to refer to PMP and has a CFG register and an ADDR register to implement the address range constraints.
(2) The master device input to IOPMP has a Source ID (SID), and IOPMP will complete the MD permission reading according to the SID. In order to reduce the resource consumption of IOPMP and bus latency, the IOPMP designed in this project can complete the indexing of up to four SIDs, while there are four different storage fields, i.e., MD0–MD3.
(3) When the MD authority of the corresponding SID is read, IOPMP will process the table entries under the corresponding MD in parallel and determine whether the request address is hit or not. If it hits, the request address is valid, and the signal output is completed according to the AHB bus protocol. Otherwise, an exception interrupt signal is generated to indicate illegal access. Since IOPMP can be cascaded for more fine-grained security access control, it can be extended by cascading multiple IOPMPs when a single IOPMP is not enough to constrain the security scope.

#### 3.5.2. Design of Mailbox

**Figure 5:**
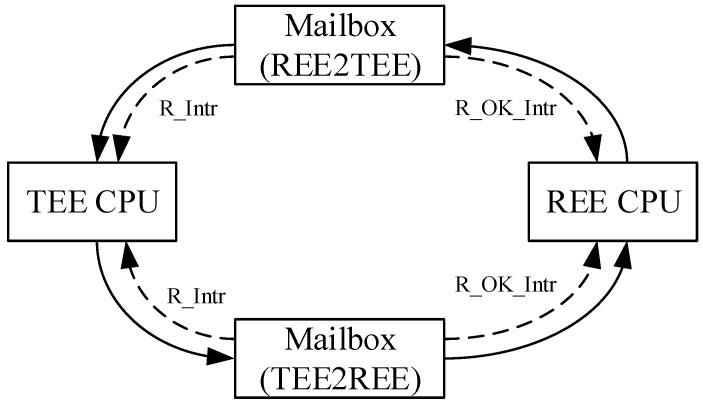
Mailbox-based dual-core communication.

In a dual-core SoC architecture, inter-processor communication (IPC) is the key to achieve data communication, event control, and resource sharing. As shown in Figure 5, DITES uses Mailbox communication based on shared memory combined with inter-core interrupts, while the shared memory is implemented using FIFO (FIFO is implemented using RegFile under the process library during ASIC synthesis). The dual-core processes can transfer data through Mailbox, i.e., read and write communication according to the mutually statutorily agreed interaction protocol. To solve the read/write consistency problem of the shared memory and protect the data security of the security core in the shared area, the TEE CPU and REE CPU use independent shared memory. As can be seen from Figure 5, when the REE CPU transfers data to the TEE CPU via Mailbox, it first writes the data type, data length, and data to the FIFO of Mailbox, and when the write is completed, the R_Intr signal will be generated to inform the TEE CPU to receive the data from Mailbox. When the TEE CPU finishes receiving the data, it generates the R_OK_Intr signal to inform the REE CPU that this communication is complete. Similarly, the transfer of data from the TEE CPU to the REE CPU is also done by the R_Intr and R_OK_Intr signals. Therefore, in order to implement dual-core communication, both processor cores need to add two Mailbox interrupt responses.

#### 3.5.3. Multi-Level Access Policy

To secure the dual-core communication, this paper proposes a multi-level access control strategy based on shared memory. The TEE CPU implements access control for the REE CPU based on the multi-level access control policy to protect the security of shared memory. The service process of communication between the TEE CPU and the REE CPU includes basic communication service, cryptographic service, etc., and different security services have different security levels. For the service requests initiated by the REE CPU, they should have different levels of access control policies. According to the security level of process access, this paper divides the processes accessing the security core into three security levels. The TEE CPU provides three different security access control policies, as shown in Table 2 below.

(1) Level 1, direct access policy: It does not require any access control, and the TEE CPU directly provides access services to the REE CPU, only requires the process to meet the protocol of dual-core communication.
(2) Level 2, integrity access policy: It authenticates the REE CPU process access to the security zone resources to ensure that the process code or data has not been maliciously tampered with.
(3) Level 3, confidentiality access policy: It includes validity and integrity access policy and data encryption policy and is used for the access control of high-security data, such as access to keys, the extraction of fingerprints, the extraction or change of passwords, the updating of data in the security zone, etc.

#### 3.5.4. Implementation of Confidential Access Policy

**Figure 6:**
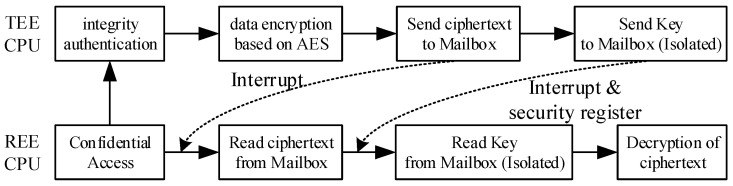
Confidential access flowchart.

We specify that the transaction requesting encryption is a confidential access event, as shown in Figure 6. For this type of access, it is first necessary to SHA1 the code block of the access task to obtain the corresponding digest and compare it with the task code digest obtained by secure boot (described in the next subsection) to achieve integrity authentication. When the authentication is passed, the TEE CPU transmits the ciphertext (using AES encryption) through the common shared memory in Mailbox; at this time, if a malicious process intercepts the value read by Mailbox, it is invalid. To transfer the key to the REE CPU, we use the security registers and the TEE-only writable memory area in Mailbox for the secure transfer. When the REE CPU finishes receiving the cipher text, the TEE CPU writes the encryption key to a separate block of memory (FIFO) in the TEE, while the write security register is valid and writes an interrupt signal to inform the REE CPU to read it. The interrupt handler of the REE CPU will first determine whether the value of the security register is valid, i.e., give the REE CPU permission to read the memory area in the Mailbox that only the TEE CPU has write access to. When it is valid, the REE CPU reads the data from the isolated memory; otherwise, it reads from the shared memory. Therefore, for confidential access, the original access process returns the value as ciphertext. The corresponding task obtains the acquisition key so that the attacker can not easily obtain the key to decrypt the ciphertext.

### 3.6. Secure Boot

**Figure 7:**
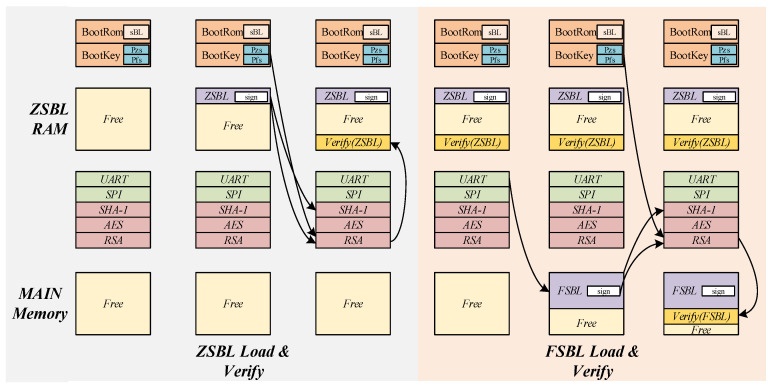
Secure boot.

Secure boot is a necessary design requirement for a TEE SoC and is a prerequisite for ensuring system security. In the secure boot process, it is usually necessary to verify the authenticity and integrity of the code to ensure that the code has not been tampered with. The secure boot process designed in this paper is shown in Figure 7. The dual-core boot process is divided into two phases: the TEE CPU boot and the REE CPU boot. The REE CPU is hung until the code is loaded and verified. The secure boot process of the TEE SoC is as follows.

(1) When the SoC is powered on and reset, the TEE CPU first runs the code in BootRom. The code is mainly responsible for loading the ZSBL code and taking the corresponding signature to the ZSBL RAM in the Isolated System through the secure serial port.
(2) The TEE CPU performs a SHA-1 extraction digest of the loaded program in the ZSBL RAM and performs the decryption of the signature using the RSA public key of the ZSBL code segment inside the BootRom for the signature verification of the loaded program. The TEE CPU jumps to ZSBL RAM to run when the verification is passed.
(3) ZSBL first configures the IOPMP restricted access address in the SoC. Then it starts the secure serial port to load the TEE_REE code and the code signature signed by the RSA private key. After the loading is completed, the TEE CPU starts the TEE software stack after the SHA-1 calculation is performed to extract the program digest and verify the signature using RSA.
(4) The TEE CPU performs SHA-1 on specific processes in the REE software stack to obtain the corresponding digest list, setting the basis for secure dual-core communication later. After completing the list generation, the TEE CPU will configure the REE CPU boot address and pull the reset signal of the REE CPU high to start the REE CPU. Hence, the secure boot of the TEE SoC is completed.

## 4. FPGA Test and ASIC Implementation

### 4.1. Introduction to the FPGA Test Platform

This paper uses Xilinx’s Kintex 7 XC7K325T-2FFG676I FPGA, which has 326 K logic cells; 50,950 lookup tables (LUT); 407,000 flip-flops; 840 DSP processing units; and a 16 Mbit block RAM for verification. The development board used for the verification also has two 32-bit DDR3 memories with a total size of 1 GB and a CH340 chip for USB-to-TTL conversion, which enables communicate between the PC and the FPGA using a USB cable.

### 4.2. Resource Utilization of FPGA

The dual-core SoC architecture designed in this paper uses Synopsys’ Synplify tool to synthetically generate circuit netlists and combines with Xilinx’s Vivado 2019.1 tool to generate bitstream. The hardware utilization of the SoC is shown in Table 3. It can be seen from Table 3 that the dual E902 processing cores occupy close to 45% of the resources, while the crypto core we designed occupies only the resources of one processor core, wherein RSA encryption and decryption are optimized to account for only about 10% of the overall resources. From the table, we can also see that the BRAM occupies a larger amount of resources (88.31% of the FPGA chip), mainly because the SoC does not use off-chip storage resources and because all programs are stored on-chip. Therefore, our future work will combine off-chip Flash and DDR to optimize the storage requirements and reduce the on-chip resource occupation.

### 4.3. Performance Analysis of Crypto Core

**Figure 8:**
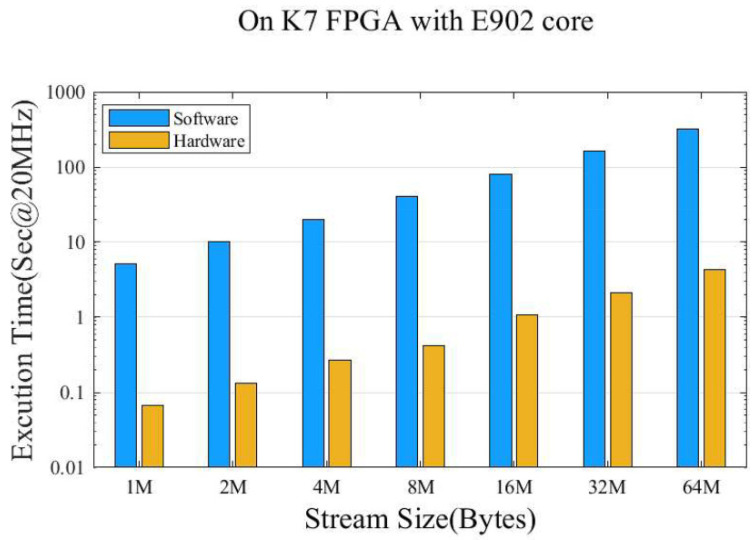
Runtime of SHA1 software and hardware.

During the process of secure boot, the speed of extracting program hash values directly affects the efficiency of program verification. Therefore, we verified the performance of a SHA1 hardware unit by performing hash digest calculation on 1 M~64 M byte streams. Code blocks of different sizes were sent to a fixed piece of memory through the UART, and then the SHA1 calculation was initiated. After the calculation was finished, the clock cycles spent for the calculation could be obtained through the counter set in SHA1, so that the time spent for the overall SHA1 calculation could be calculated, and the clock frequency of the SoC in this design was 20 MHz. Meanwhile, the same timing method was used for software calculation based on E902; the run time of both is shown in Figure 8, and the acceleration ratio can reach 75. Meanwhile, we also tested the RSA algorithm responsible for the signature and verification tasks during the secure boot process. As can be seen from Table 4, the RSA algorithm designed in this paper does not significantly increase computation time with the addition of the random modulus multiplication operation. In the secure boot, the verification (decryption) was performed. In our design, the decryption time of the 192 bit key was only 61.7 us, which means that 16,207 decryption tasks could be completed every second. As shown in Table 4, we also compared the encryption and decryption times under different RSA implementations. When the key length was 1024 bit, our RSA computing unit’s decryption and encryption speed was 1331 and 1493 times faster than E-RSA. Furthermore, for the higher-performance desktop-class processor Intel i9-10850k, our designed RSA unit’s decryption and encryption time was only 0.16 and 0.14 of P-RSA when the RSA key length reached 2048 bit. As a result, the crypto core not only had improved performance but also better flexibility.

### 4.4. Dual-Core Performance Test

This paper builds a multi-stage bus architecture, which is based on the principle of AHB bus distribution. For dual-core operation, i.e., for the second bus level of this architecture, there are two master devices accessing the bus slave devices and are thus bound to cause single-core processing performance loss. In this paper we use Coremark and Dhrystone to test single-core and multi-core performance, and we use Coremark’s 2 K performance running parameters, while Dhrystone’s test count is 1 million, and the test results are shown in Table 5. As seen in Table 5, for the Coremark test, the single-core performance is 2.21 Coremarks/MHz. but when the dual-core is running at the same time, the TEE CPU performance loss reaches about twice the REE CPU. For the Dhrystone test, the single-core E902 score results can reach 1.36 DMIPS/MHz, which is already equivalent to an average processor. When the dual cores are booted and run simultaneously, the TEE CPU performance is reduced to 0.47 DMIPS/MHz, while the REE CPU performance loss is smaller, reaching 0.92 DMIPS/MHz. In single-core scenario, since the E902 is primarily oriented to computational tasks for embedded applications, its speed is 0.213 DMIPS/MHz lower than the Rocket for high-performance computing, but the Rocket uses 4.4 times more logical resources than the E902, occupying a larger area resource, as shown Table 6. Compared to IBex with RV32 ISA, the speed of the TEE with dual-core is still 0.036 DMIPS/MHz higher than IBex. As analyzed in Table 5, our designed SoC architecture has a hierarchical bus, resulting in a large reduction in the efficiency of the highest level of processing access, but for real-world applications, the TEE CPU is only responsible for a few corresponding security tasks after the security startup, while the REE CPU handles the main computational tasks, so the architecture designed in this paper does not significantly affect the efficiency of the SoC.

### 4.5. Comparison of TEE SoCs

Current researchers in the implementation of TEE SoCs have proposed different architectures for their designs. The comparison between the work in this paper and similar TEE SoC platforms is shown in Table 6. For CURE [14] and HECTOR-V [15], their architectures do not integrate cryptographic algorithm hardware computation units, which will lead to a less efficient system secure boot, while for [9,10,11], the core CPU used is based on open-source Rocket, which is mainly oriented for high-performance computational tasks; thus it consumes more hardware logic resources, which is nearly four times more compared to the open-source E902 used in this project. Furthermore, more resources will inevitably lead to higher power consumption. Therefore, it is not suitable to be applied to resource- and power-sensitive edge devices. Meanwhile, the hardware encryption and decryption unit designed in our TEE SoC consumes less power and resources. Compared with the design using the elliptic curve algorithm, the encryption core designed in this paper can realize AES encryption and SHA digest reading and RSA encryption and decryption with multiple key bit widths, which makes the whole TEE system more flexible.

The level of support for each security feature on different platforms is listed at the bottom of Table 6, where the security features are described as follows. **Secure Boot** indicates whether the TEE SoC is designed to support secure boot; **Flexible Boot** indicates whether the code loaded in Secure Boot can be flexibly modified; **Exclusive TEE CPU** indicates whether it has a separate TEE; **TEE Isolation** indicates whether the current architecture supports a fully isolated environment for TEE security cores; **Isolated Storage** indicates whether the current architecture supports isolated storage for secure operation; **SCA Protection** indicates whether the current design supports resistance to side-channel attack protection; **Secure IPC** indicates whether the multi-core SoC is capable of secure communication; **Hardware Cost** indicates the resource utilization of the architecture.

DITES supports SHA1 digital digest extraction, AES128/256, and RSA with five different key lengths, enabling secure boot and flexible boot configurations. The SoC architecture designed in this paper has implemented the first level of security isolation bus, completely isolating the exclusive TEE CPU from the REE world and ensuring the security of data. Furthermore, the TEE tasks run independently on the TEE CPU to achieve TEE isolation. Meanwhile, we designed a multi-level access policy to ensure the security of inter-core communication and used a fully isolated SRAM under the TEE first-level bus for storing secure buffer data to achieve the purpose of Isolated Storage. We also added a pseudo-random calculation into RSA operation to protect the information from being easily accessed during encryption and decryption process. Then, we use low-power and low-cost processor, so DITES works with lower hardware costs. Thus, the proposed DITES has better SCA protection and secure IPC, that is, our designed TEE SoC system can provide lower resource consumption, higher flexibility, and better security.

### 4.6. ASIC Implementation

To further validate our SoC design architecture, we use Synopsys’ Design Compiler (DC) to synthesize the TEE SoC using the TSMC 90 nm library, and the synthesis results are given in Table 7. Since the SoC architecture proposed in this paper uses on-chip SRAM as the main memory, the memory occupies a larger area. As can be seen from the table, the number of gates occupied by dual-core processors and cryptographic units under this architecture is small, reaching only 17.4%. Furthermore, for IOPMP, which restricts access rights, and Mailbox, which enables dual-core communication, only 1.25% and 1.02% are occupied. As for the power consumption, the overall power consumption of this architecture is only 0.048 W, of which the power consumption of the dual core accounts for 60% of the total, while the power consumption of the acceleration unit is only about 10%. Meanwhile, moving most of the memory off-chip reduces chip area and saves more static power.

## 5. Limitations and Future Work

First, as analyzed in Section 4.4, the dual-core shared bus in our system leads to some performance loss. To improve the performance of DITES, we suggest two possible methods. One is to choose a RISC-V processor with higher performance. The second is to optimize the storage domain architecture and physically isolate the TEE program and the REE program, improving the access efficiency of the dual-core processor.

Second, our system supports encryption and decryption algorithms, i.e., RSA, AES, and SHA1. To further improve the security and application scope of DITES, we will continue to extend more cryptographic computational units on the SoC architecture, such as true random number generation (TRNG), elliptic curve-based encryption and decryption (Ed25519), and digest extraction (SHA3).

Finally, as shown in the DC synthesis results, DITES is similar to the literature [9], i.e., it occupies a large storage area. Therefore, in the future, we will consider the main memory and related storage to be implemented using off-chip memories, such as DDR and Flash, which can reduce the chip area.

## 6. Conclusions

In this paper, a TEE-secure SoC system with a multi-level bus architecture based on a dual-core CPU for RISC-V is proposed. The newly proposed IOPMP of RISC-V is also utilized to restrict the access rights of CPUs in a non-secure environment. In order to improve the processing efficiency of secure boot and encryption and decryption operations, we designed hardware computing units for RSA, AES, and SHA1 algorithms and integrated them into the TEE SoC as a crypto core, and IOPMP restricts its access rights. At the same time, we designed a simple secure boot process based on the CoT to ensure the security of the SoC environment boot. We use Mailbox based on shared memory and inter-core interrupts to enable dual-core communication. We have performed FPGA prototype verification and DC synthesis of the designed TEE SoC. Based on the experimental results, we can know that our architecture has lower resource consumption, higher flexibility, and better security, which can well meet the basic security and computing requirements of IoT edge devices.

## Figures and Tables

**Table 1:** A Review of Related Hardware Implementations of TEE.

Refs.	Year	Architecture	Cryptography	Complexity	Flexibility	Secure IPC	DC Library	Focus
[9]	2022	Dual Core (Rocket)	SHA3, AES, Ed25519	High	Medium	N/A	ROHM −180 nm	TS
[10]	2020	Dual Core (Rocket)	SHA3, Ed25519	High	Low	N/A	N/A	CA
[11]	2019	Dual Core (Rocket)	CAU + KMU	High	Low	N/A	N/A	TS
[12]	2019	Dual Core (Rocket)	CAU + KMU	High	Low	N/A	N/A	Boot
[14]	2021	Dual Core (Rocket)	N/A	High	Low	N/A	N/A	SA
[15]	2021	Single Core (lowRISC)	N/A	Medium	Low	N/A	N/A	TS
[17]	2021	CPU-FPGA	ECDH, PUF	Medium	Low	N/A	N/A	TS
[18]	2021	Single Core (lowRISC)	ChaCha	Medium	Low	N/A	N/A	MEU
[19]	2020	CPU-FPGA	PUF	Medium	Low	N/A	N/A	TS
Our	2022	Dual Core (E902)	RSA, AES, SHA1	Low	High	Multi-level Access	TSMC 90 nm	TS

TEE Env: TEE Environment; TS: TEE System.

**Table 2:** Policy for shared memory access.

Security Level	Security Policy	Access Authentication	Security	Flexibility	Efficiency
Level 1	Direct access	NO	Low	High	High
Level 2	Integrity Authentication policy	Integrity authentication	Medium	Medium	Medium
Level 3	Encrypted authentication policy	Integrity authentication, data encryption	High	Low	Low

**Table 3:** Dual-core SoC in Kintex 7 XC7K325T Hardware Utilization.

Paras.	Dual CPU		Crypto Core			Mailbox	IOPMP	Total
	TEE	REE	RSA	AES	SHA1			
**LUTs**	8529	8185	3963	2656	1399	172	3889	37,484
**Registers**	2696	2292	3933	1283	1608	182	1211	17,272
**BRAM**	0	0	7	0	0	1	0	393
**DSP**	0	0	4	0	0	0	0	4
**Utilization (%)**	22.75	21.83	10.57	7.09	3.73	0.46	10.37	100
**Power**	0.005	0.006	0.022	0.013	0.002	0.001	0.001	0.297

**Table 4:** RSA encryption and decryption times with different key lengths.

Method		Key Length/bit				
		192	256	512	1024	2048
**RSA**	**Decryption**	61.7 us	90.8 us	273.4 us	961.4 us	3.6 ms
	**Encryption**	843.31 us	1.6 ms	9.7 ms	68.6 ms	527.4 ms
**Random** **RSA**	**Decryption**	84.7 us (+0%)	128.7 us (+52%)	375.3 us (+192%)	1.3 ms (+246%)	4.8 ms (+269%)
	**Encryption**	983.4 us (+0%)	1.90 ms (+93%)	11.6 ms (+510%)	80.3 ms (+592%)	613.8 ms (+664%)
**E-RSA**	**Decryption**	335 ms	446.8 ms	860.7 ms	1.73 s	3.98 s
	**Encryption**	4.30 s	7.61 s	31.55 s	119.93 s	500.73 s
**P-RSA**	**Decryption**	994.44 us	2.99 ms	4.98 ms	12.96 ms	28.92 ms
	**Encryption**	22.94 ms	43.88 ms	192.48 ms	916.54 ms	4.22s

E-RSA: RSA running on an E902; P-RSA: RSA running on an Intel i9-10850k using python.

**Table 5:** Test results of Coremark and Dhrystone.

Core		ISA	Coremark Test	Dhrystone Test	
			Coremarks/MHz	Dhrystone/s	DMIPS/MHz
**Single-core**		**RV32EMC**	2.21	47,619	1.36
**Dual-core**	**TEE CPU**		1.04 (−1.17)	16,666	0.47 (−0.89)
	**REE CPU**		1.69 (−0.52)	32,258	0.92 (−0.44)
**[8]**	**Rocket**	RV32IMC	N/A	138,197	1.573
	**IBex**	RV32IMC	N/A	38,165	0.434

**Table 6:** Comparison of similar TEE SoCs.

Paras.		[14]	[15]	[11]	[9]	[10]	This Work
**Architecture (Core)**		Dual	Single	Dual	Dual	Dual	Dual
**Core**	**Name**	Rocket	lowRISC	Rocket	Rocket	Rocket	E902
	**LUTs**	74,258	55,443	74,258	74,258	161,678	16,714
**Crypto** **Core**	**Yes/No**	No	No	Yes	Yes	Yes	Yes
	**Utilization**	—	—	+27,170 (36.59%)	+19,883 (26.77%)	+14,642 (+9.06%)	+8018 (47.97%)
**Security features in TEE SoC**							
**Secure Boot**		**☆☆**	**☆☆☆**	**☆☆☆**	**☆☆☆**	**☆☆**	**☆☆☆**
**Flexible Boot**		**☆☆☆**	**☆☆☆**	**☆**	**☆☆☆**	**☆☆**	**☆☆☆**
**Exclusive TEE CPU**		**☆☆**	**☆☆☆**	**☆**	**☆☆☆**	**☆**	**☆☆☆**
**TEE Isolation**		**☆**	**☆**	**☆**	**☆**	**☆**	**☆☆☆**
**Isolated Storage**		**☆**	**☆☆☆**	**☆☆☆**	**☆☆☆**	**☆☆☆**	**☆☆☆**
**SCA Protection**		**☆☆☆**	**☆☆☆**	**☆**	**☆**	**☆**	**☆☆**
**Secure IPC**		**☆**	**☆**	**☆**	**☆**	**☆**	**☆☆☆**
**Hardware Cost**		**☆**	**☆**	**☆**	**☆☆☆**	**☆☆**	**☆☆☆**

**☆☆☆**, **☆☆**, and **☆** represent the best to the worst support level.

**Table 7:** The synthesis result-based TSMC 90 nm.

Paras.	Cell-Count (NAND2)	Cell-Area		Power			
		um^2^	%	Leakage (uW)	Dynamic (uW)	Total (uW)	%
**Total**	1,799,804	5,075,448	100	17,768	24,309.9	48,828.1	100
**TEE Core**	43,796	123,507	2.43	247.79	13,978.1	14,225.9	29.13
**REE Core**	43,796	123,507	2.43	247.79	13,978.1	14,225.9	29.13
**RSA**	173,522	489,334	9.64	1474.4	2056.1	3530.6	7.23
**AES**	36,785	103,734	2.04	200.73	944.1777	1144.9	2.34
**SHA1**	15,444	43,553	0.86	90.30	445.1427	535.5	1.10
**Mailbox**	18,325	51,768	1.02	905.82	44.53	950.4	1.95
**IOPMP**	22,412	63,202	1.25	91.99	391.8656	483.9	0.99
**Memory**	1,544,254	4,354,798	85.80	16,357	1.837	16,790.7	34.39

## Data Availability

Not applicable.

## Abbreviations

AI	Artificial Intelligence
TEE	Trusted Execution Environment
SoC	System-on-Chip
CPU	Central Processing Unit
IPC	Inter-processor Communication
RISC-V	Reduced Instruction Set Computer-V
SHA1	Secure Hashing Algorithm-1
AES	Advanced Encryption Standard
RSA	Rivest–Shamir–Adleman
IOPMP	Input/Output Physical Memory Protection
CoT	Chain-of-Trust
AHB	Advanced High-Performance Bus
ISA	Instruction Set Architecture
RISC-V	Reduced Instruction Set Computer-V
FPGA	Field-Programmable-Gate-Array
ASIC	Application-Specific Integrated Circuit
ZSBL	Zero Stage Boot Loader
MEU	Memory Encryption Unit
MCU	Microcontroller Unit
